# ASPP2 Plays a Dual Role in gp120-Induced Autophagy and Apoptosis of Neuroblastoma Cells

**DOI:** 10.3389/fnins.2017.00150

**Published:** 2017-03-24

**Authors:** Zhiying Liu, Luxin Qiao, Yulin Zhang, Yunjing Zang, Ying Shi, Kai Liu, Xin Zhang, Xiaofan Lu, Lin Yuan, Bin Su, Tong Zhang, Hao Wu, Dexi Chen

**Affiliations:** ^1^Center for Infectious Diseases, Beijing Youan Hospital, Capital Medical UniversityBeijing, China; ^2^Beijing Institute of Hepatology, Beijing Youan Hospital, Capital Medical UniversityBeijing, China; ^3^Organ Transplantation Center, The Affiliated Hospital of Qingdao UniversityQingdao, China

**Keywords:** HAND, autophagy, ASPP2, gp120

## Abstract

HIV invasion of the central nervous system (CNS) in the majority of patients infected with HIV-1, leads to dysfunction and injury within the CNS, showing a variety of neurological symptoms which was broadly termed HIV-associated neurocognitive disorder (HAND). But the molecular mechanisms are not completely understood. It has been suggested that apoptosis and autophagic dysfunction in neurons may play an important role in the development of HAND. Previous studies have indicated that p53 may be involved in the onset of neurological disorder in AIDS. Apoptosis-stimulating protein of p53-2 (ASPP2), a p53-binding protein with specific function of inducing p53, has been reported to modulate autophagy. In the present study, we observed that gp120 induces autophagy and apoptosis in SH-SY5Y neuroblastoma cells. Adenovirus-mediated overexpression of ASPP2 significantly inhibited autophagy and apoptosis induced by low dose of gp120 protein (50 ng/mL), but induced autophagy and apoptosis when treated by high dose of gp120 protein (200 ng/mL). Further, ASPP2 knockdown attenuated autophagy and apoptosis induced by gp120.

**Conclusion:** ASPP2 had different effects on the autophagy and apoptosis of neurons induced by different concentration of gp120 protein. It may be a potential therapeutic agent for HAND through modulating autophagy and apoptosis in CNS.

## Introduction

HIV-1-associated neurocognitive disorder (HAND), also known as neuroAIDS, occurs in one-third of HIV-1-infected individuals (Gendelman et al., 1997). Symptoms range from minor cognitive difficulty to severe dementia. Both HAND and HIV-1-associated dementia (HAD) result in serious impacts on the quality of life and prognosis. Although highly active anti-retroviral therapy (HAART) has decreased the incidence of HAND, as the extension of lifespan, the prevalence of HAND is actually increasing (Boisse et al., 2008) in HIV-infected population.

Autophagy executes an essential role in growth regulation and cellular homeostasis through delivering aging proteins and organelles to lysosomes for degradation (Klionsky et al., 2012). A series of proteins participate in autophagy. Beclin1, which can interact with Bcl-2 to form a complex, plays a critical role for the initiation and the late stage of autophagy (Liang et al., 1998, 1999; Pattingre et al., 2005; Zeng et al., 2006). Microtubule-associated protein light chain 3 (LC3) forms a stable association with the membrane to initiate formation and lengthening of the autophagosome. LC3 exists as two forms: LC3-I which localizes in the cytoplasm and LC3-II localize and binds to membrane, and its conversion from LC3-I to LC3-II is considered as specific marker of autophagy (Kabeya et al., 2000, 2004; Asanuma et al., 2003).

The dysfunction of autophagy has been reported in several neurodegenerative diseases including Alzheimer's, Parkinson's (Pan et al., 2008), and Huntington's diseases (Petersen et al., 2001; Pickford et al., 2008) as well as in advanced AIDS (Alirezaei et al., 2008; Zhou et al., 2011). HIV may interfere with autophagy by various viral proteins (Espert et al., 2006, 2009). Postmortem brains with HIV-1 encephalitis exhibit increased autophagic proteins such as lysosomal membrane protein 1 (LAMP-1), autophagy-related gene (Atg)-5, Atg-7, Beclin 1, and LC3II when compared with the brains from HIV-infected persons without HIV-1 encephalitis or the control brains without HIV-infection, suggesting that the dysregulation of autophagy may be important in the pathogenesis of neuroAIDS and plays a particularly important role in the early cognitive impairment and dementia in advanced AIDS (Zhou et al., 2011).

Gp120 is a soluble envelope glycoprotein inducing apoptosis through interaction with chemokine receptors such as CXCR4 and CCR5. It's is a potential HIV neuronal toxic protein which caused neuronal loss and dendritic simplification (Toggas et al., 1994). Many studies have reported that gp120 can induce neuronal apoptosis. However, the mechanism of neurotoxicity induced by gp120 through directly interacting with neurons or indirect effects (through inflammatory responses) is not determined yet. It was reported that p53 was required in the apoptosis induced by gp120 in neurons (Yeung et al., 1998).

Apoptosis-stimulating protein of p53-2 (ASPP2) is a p53-binding protein that specifically stimulates pro-apoptosis of p53 (Samuels-Lev et al., 2001), which plays a key role in gp120-mediated neurotoxicity (Garden et al., 2004). Under stress condition such as hypoxia, ionizing radiation, DNA damage, and chemotherapeutic drugs (Patel et al., 2008), ASPP2 binds to nuclear P53, leading to the transcription of p53-targeted pro-apoptosis gene such as Bax and p53-upregulated-modulator-of-apoptosis (PUMA). ASPP2 can also regulate autophagy in cancers (Wang Y. et al., 2013). However, its effects on apoptosis induced by gp120 are rarely studied. In this study, we hypothesize that ASPP2 plays an important role in gp120 mediated neurotoxixity. To determine it, we investigated the autophagy and apoptosis induced by different gp120 concentrations in neuroblastoma cells. We found that ASPP2 could be involved in the gp120 mediated neurotoxixity, potentially via modulating apoptosis and autophagy.

## Materials and methods

### Cell culture and treatment

SH-SY5Y neuroblastoma cells were grown in Dulbecco's Modified Eagle's medium (DMEM) supplemented with 10% fetal bovine serum (FBS), 100 units/mL penicillin, and 100 μg/mL streptomycin (Invitrogen Life Technology, USA) at 37°C and 5% CO_2_. To examine the effect of ASSP2 on autophagy and apoptosis induced by gp120 (Abcam, USA), SH-SY5Y neuroblastoma cells were transduced with adenovirus overexpressing ASPP2 with GFP flag (GFP-ASPP2-rAd) or GFP (GFP-rAd) for 36 h. Cells were then treated with gp120 and apoptosis and autophagy were analyzed 24 h later. In a parallel study, SH-SY5Y neuroblastoma cells were transfected with ASPP2 siRNA or scrambled siRNA (target sequence: 5′-UAUGCAGAGACGUGGUGGATT-3′ and scrambled siRNA: UUCUCCGAACGUGUCACGUTT), and apoptosis and autophagy were analyzed same as described above.

### Analysis of cell viability and apoptosis

Cell viability was analyzed by Calcein/PI kits (Sigma, Germany). In brief, cells were incubated with 1 mg/mL Calcein/AM for 30 min and then added PI at 10 mg/mL for 15 min. Cells were washed and cell viability was analyzed with an inverted microscope (Nikon Eclipse TE200). Cells were counted in a blinded manner and presented as the ratio of PI-positive cells/(Calcein positive + PI positive cells). Each experiment was replicated three times with three randomly selected fields per well and three wells per condition. Cell viability was also measured by flow cytometric analysis in selective experiments using AnnexinV/7-aad kit (Southern Biotech, USA) according to manufacturer's instructions.

### Western blot and immunoprecipation (IP)

Cells were collected, washed with PBS, and then lysed with lysis buffer (150 mM NaCl, 1% NP-40, 0.5% deoxycholate, 0.1% SDS, 50 mM Tris (pH 8.0, and 5 mM EDTA) containing protease inhibitors (10 μg/mL phenylmethylsulfonyl fluoride). After quantification with BCA assay (Beyotime Biotechnology, Beijing, China), 30 μg total proteins were loaded and separated on 10% SDS-PAGE gel and transferred to PVDF membrane. The membrane was blocked with 5% non-fat milk, probed with rabbit polyclonal antibodies against human LC3 or cleaved LC3 (LC3-II) or Beclin 1 (Cell Signaling Technology, USA), mouse monoclonal antibody against human β-actin (Cell Signaling Technology, USA), and ASPP2 (sigma, Germany). The membranes were washed, probed with HRP conjugated secondary antibody, and detected with chemiluminescent kit. The western blot images were quantitatively analyzed with Image J software (National Institutes of Health, USA).

### Quantification of GFP-LC3 puncta

To observe LC3 puncta, plasmids pCMV-GFP-LC3 which contains LC3 fused with tracer GFP and pCMV-ASPP2 was transfected into SH-SY5Y neuroblastoma cells with Effectene Transfection Reagent (QIAGEN, Germany) for 24 h, and then treated with gp120 protein at various concentrations. Cells were fixed 24 h later and nuclei were stained with 4,6-diamidino-2-phenylindole (Invitrogen, USA). GFP-LC3-labeled autophagosomes were imaged and quantitatively counted. The data was presented as % of GFP-LC3 positive cells.

### Statistical analysis

All data were expressed as Mean ± SEM from three independent experiments. Statistical analysis was performed using SPSS 16.0 by non-parametric Kruskall-Wallis and Mann-Whitney U test. *P* < 0.05 was considered as statistically significant.

## Results

### gp120 dose-dependently induced autophagy and apoptosis in SH-SY5Y neuroblastoma cells

In our previous study, we found that the expression and location of p53 and ASPP2 could change with different gp120 treatment concentrations in mice cerebrocortical neurons (Liu et al., 2016). Thus, we also tested two different concentrations (50 and 200 ng/mL) of gp120 at the present study. In addition, These two gp120 concentrations used here have been indicated to have physiological relevance in some other studies (Louboutin and Strayer, 2012; Pandhare et al., 2015). SH-SY5Y is a human neuroblastoma cell line expressing N-methyl-D-aspartic acid (NMDA) receptors, CXCR4, CCR5, and but not CD4, and can binds to gp120 proteins. To prove whether gp120 induces autophagy and apoptosis, SH-SY5Y cells were treated with gp120 at the doses of 50 and 200 ng/mL for 24 h and autophagic marker LC3-II and Beclin 1 were analyzed with western blot analysis and immunohistochemical staining.

**Figure 1:**
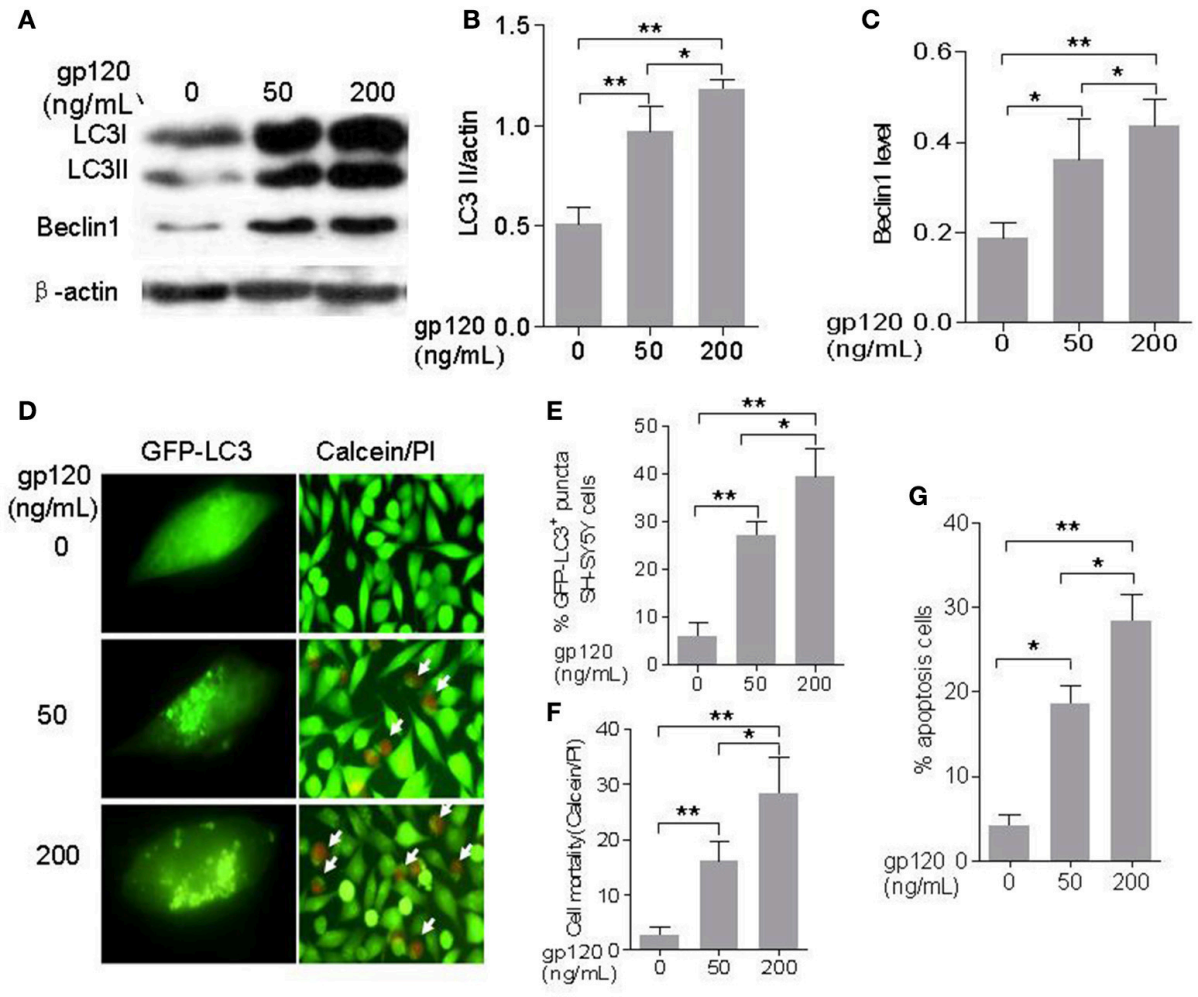
**HIV-1 gp120 can induce autophagy and apoptosis in SH-SY5Y neuroblastoma cells. (A)** Bands of autophagic proteins of LC3-I/LC3-II, Beclin1, and beta-actin treated by different concentrations of HIV-1 gp120. **(B)** Data of the LC3-II/actin. **(C)** Beclin1 were normalized with beta-actin and analyzed with ImageJ software (National Institutes of Health). **(D)** Autophagosomes formation in SH-SY5Y cells treated by HIV-1 gp120 with different concentrations (100× objective lens) and cell viability of SH-SY5Y cells treated by HIV-1 gp120 with different concentrations (cells with green color were alive and cells with red color were dead or apoptosis; 40× objective lens). **(E)** The mean levels of GFP-LC3 positive puncta with different treatments. **(F)** The mean levels of cell mortality with different treatments by Calcein AM/PI kit. **(G)** The mean levels of apoptosis cells with different treatments by Annexin V/7-aad kit. All data were shown as the mean ± S.E.M. of three independent experiments. ^*^P < 0.05; ^**^P < 0.01.

As show in Figures 1A–C, gp120 can significantly increase the levels of Beclin1 and LC3-II at a dose-dependent manner. The relative level of LC3-II (normalized with actin) was 0.97 ± 0.07 fold (*p* < 0.05) at the dose of 50 ng/mL and increased to 1.18 ± 0.03 fold (*p* < 0.01) at the dose of 200 ng/mL as compared with the control group. Beclin1 level was 0.31 ± 0.02 (*p* < 0.05) at the dose of 50 ng/mL and increased to 0.45 ± 0.02 fold (*p* < 0.05) at the dose of 200 ng/mL. Cells with GFP-LC3 positive puncta also significantly increased from 27 ± 1.6% (*p* < 0.05) at the dose of 50 ng/mL to 39 ± 3.38% (*p* < 0.05) at the dose of 200 ng/mL (Figures 1D,E). The cell death, as determined with calcein/PI, increased from 16 ± 2.08% at dose of 50 to 28.3 ± 3.75% (*p* < 0.05) at dose of 200 (Figures 1D,F). Similarly, apoptotic cells increased from 18.6 ± 1.28% at the dose of 50 ng/mL to 28.3 ± 1.85% (*p* < 0.05) at the dose of 200 ng/mL (Figure 1G, Figure S1). These findings indicated that gp120 could induce autophagy and apoptosis in SH-SY5Y neuroblastoma cells at a dose-dependent manner.

### ASPP2 overexpression inhibited autophagy at low dose gp120 (50 ng/ml) treatment and induced autophagy at high dose gp120 (200 ng/ml) treatment in SH-SY5Y cells

**Figure 2:**
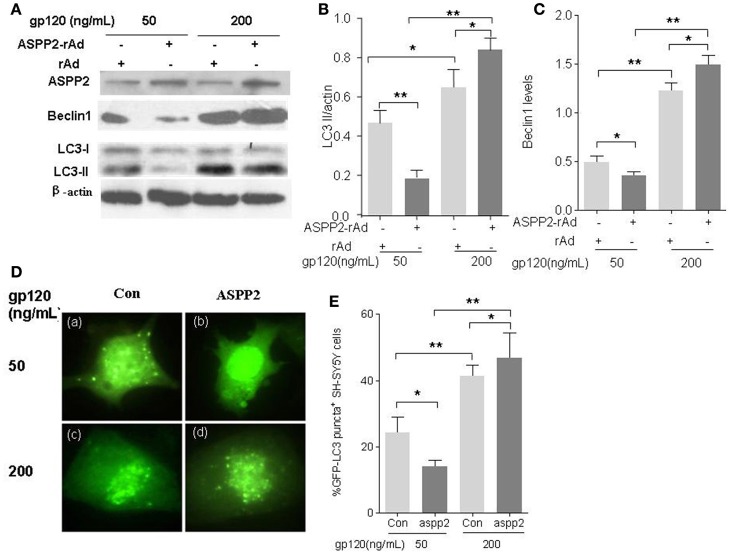
**Overexpression of ASPP2 inhibits autophagy in SH-SY5Y neuroblastoma cells when treated by 50 ng/mL gp120 but promotes autophagy when treated by 200 mg/ml gp120. (A)** Bands of ASPP2, Beclin1, LC3-I/LC3-II of SH-SY5Y cells which were infected with ASPP2-rAd and the control rAd for 36 h then treated by 50 and 200 ng/mL gp120, respectively. **(B)** Data of the LC3-II/actin. **(C)** Relative levels of Beclin1 normalized with beta-actin. **(D)** The detection of cells with GFP-LC3 positive puncta when treated by 50 and 200 ng/mL gp120, respectively. Representative images are shown (with 100× objective lens). **(E)** The mean levels of GFP-LC3 positive puncta cells treated with different concentrations of gp120. A total of 100 cells were counted in each group. All data were shown as the mean ± S.E.M. of three independent experiments, ^*^p < 0.05, ^**^p < 0.01.

In order to explore the effect of ASPP2 on autophagy of SH-SY5Y cells induced by gp120, SH-SY5Y cells were infected by green fluorescent protein-labeled ASPP2 recombinant adenovirus (GFP-ASPP2-rAd) and the control recombinant adenovirus GFP-rAd for 36 h, respectively, and then treated with gp120 protein at the dose of 50 and 200 ng/mL for 24 h, respectively. Our results indicated that the overexpression of ASPP2 could inhibit the LC3-II and Beclin1 levels at 50 ng/mL, but enhance the LC3-II and Beclin1 levels at 200 mg/mL in contrast. When treated by low dose of gp120 (50 ng/mL), the LC3-II level in ASPP2-ad group (0.19 ± 0.03) was significantly less than the rAd group (0.47 ± 0.05, *p* < 0.01). However, when SH-SY5Y cells were treated by 200 ng/mL gp120, the LC3-II level in ASPP2-ad group (0.84 ± 0.04) was significantly higher than the rAd group (0.65 ± 0.06, *p* < 0.05; Figures 2A,B). The Beclin1 level was also significantly decreased when treated by 50ng/mL gp120 in ASPP2-ad group (0.35 ± 0.02 vs. 0.49 ± 0.03 in rAd control group, *p* < 0.05), but was significantly increased when treated by 200 ng gp120 in ASPP2-ad group compared to the rAd group (1.49 ± 0.04 vs. 1.22 ± 0.03, *p* < 0.05; Figures 2A,C).

To further confirm the role of ASSP2 in autophagy, pCMV-ASPP2 plasmids and GFP-LC3 plasmids were co-transfected into SH-SY5Y cells, and then challenged with gp120. The immunofluorescence results showed that GFP-LC3 positive puncta in pCMV-ASPP2 group significantly less than that in control plasmids group (14 ± 1.15 vs. 24.3 ± 2.7%, *p* < 0.05) when treated by 50 ng/mL gp120. However, when treated by 200 ng/mL gp120, the population of the autophagic cells in pCMV-ASPP2 group was significantly higher than that in the control plasmids group too (46.7 ± 4.32 vs. 41.3 ± 1.74%, *p* < 0.05; Figures 2D,E). In summary, these results suggested that ASPP2 overexpression could inhibit autophagy at low dose gp120 but induce autophagy at high dose of gp120 in SH-SY5Y cells.

### The overexpression of ASPP2 could enhance apoptosis in the presence of gp120 treatment

**Figure 3:**
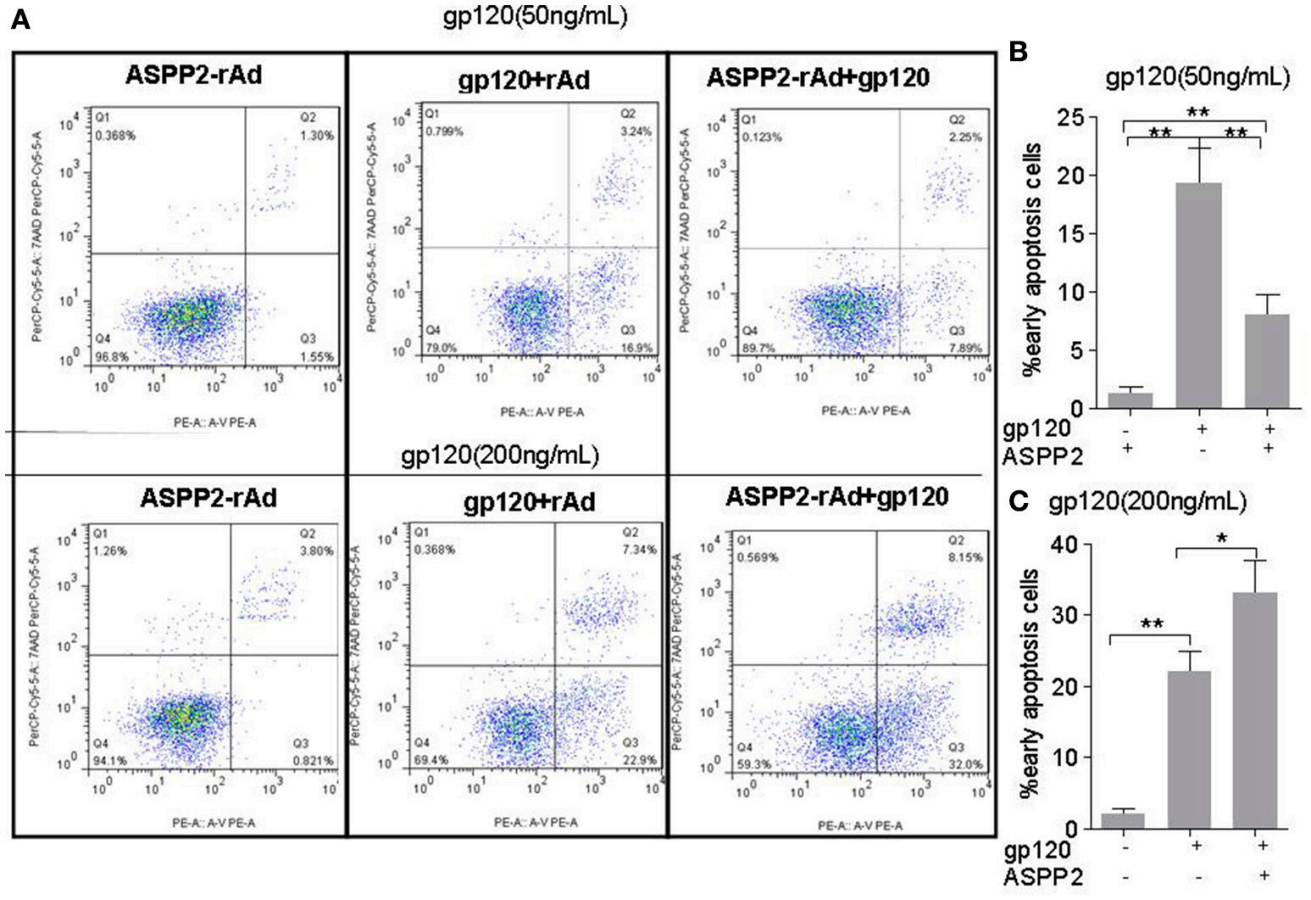
**ASPP2 overexpression enhanced apoptosis in SH-SY5Y cells when treated by 200 ng/mL gp120 but inhibited apoptosis in cells that have been treated by 50 ng/mL gp120. (A)** Representative images of early apoptosis using the flow cytometric analysis following staining with AnnexinV/7-aad kit. Cells with early apoptosis that stained with Annexin V-PE only are located in the lower right quadrant of the three displays. **(B,C)** The mean levels of early apoptotic cells in different groups. All data were shown as the mean ± S.E.M. of three independent experiments. ^*^P < 0.05; ^**^P < 0.01.

Many studies have reported that HIV-1 gp120 can induce neuronal apoptosis in which P53 plays a key role (Garden et al., 2004). Thus, we further investigated role of ASPP2 in neuronal apoptosis induced by gp120. SH-SY5Y neuroblastoma cells were infected with ASPP2-rAd for 36 h and then treated by low or high dose gp120 protein for 24 h. The apoptosis were detected by flow cytometry with AnnexinV/7-aad staining. As shown in Figures 3A,B, apoptotic cells in ASPP2-rAd group was significantly lower than that in rAd group (8.11 ± 0.42 vs. 19.36 ± 0.51%, *p* < 0.01) when treated by 50 ng/mL gp120. ASPP2-rAd itself doesn't induce apoptosis without gp120 treatment. However, apoptotic cells (Figures 3A,C) were significantly increased to 33 ± 2.65% in ASPP2-rAd group as compared with 19.3 ± 1.45% in rAd control group when treated by 200 ng/mL gp120 for 24 h (*p* < 0.05), indicating that ASPP2 overexpression could inhibit apoptosis at low dose gp120 treatment but promote apoptosis at high dose gp120 treatment instead.

### The silence of ASPP2 promoted autophagy and apoptosis in SH-SY5Y cells in the presence of low dose gp120 and decreased autophagy and apoptosis in the presence of high dose gp120

**Figure 4:**
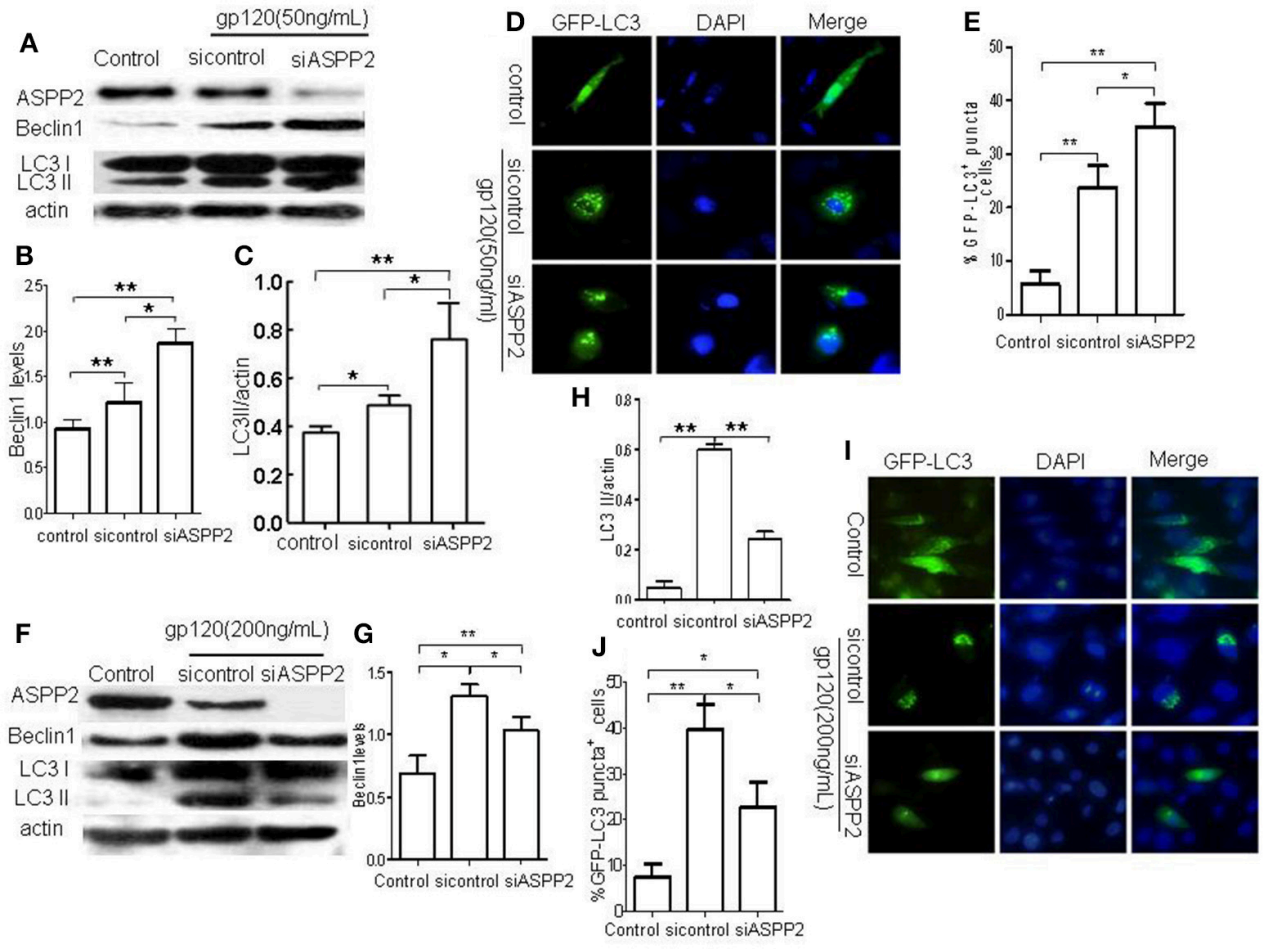
**The silence of ASPP2 promoted autophagy and apoptosis in SH-SY5Y cells in the presence of low dose gp120 and decreased autophagy and apoptosis in the presence of high dose gp120. (A)** Bands of ASPP2, Beclin1, and LC3-I/LC3-II of SH-SY5Y cells which were transfected by ASPP2 siRNA and control siRNA then treated by 50 ng/mL gp120. **(B)** Relative levels of Beclin1. **(C)** Data of the LC3-II/actin in different groups. **(D)** Detection of cells with GFP-LC3 positive puncta of autophagy in different group. **(E)** The mean levels of cells with autophagy in different groups, a total of 100 cells were counted in each group. **(F)** Bands of ASPP2, Beclin1, and LC3-I/LC3-II of SH-SY5Y cells which were transfected by ASPP2 siRNA and control siRNA then treated by 200 gp120. **(G)** Relative levels Beclin1 in different groups when treated by 200 gp120. **(H)** Data of LC3-II/actin in different groups. **(I)** Detection of SH-SY5Y cells with GFP-LC3 positive puncta of autophagy in different groups when treated by 200 ng/mL gp120. **(J)** The mean levels of autophagy cells in different groups when treated by 200 ng/mL gp120. A total of 100 cells were counted in each group and data was shown as the mean ± S.E.M of three independent experiments. ^*^P < 0.05; ^**^P < 0.01.

To further verify the role of ASPP2 in autophagy and apoptosis induced by gp120, ASPP2 was knocked down with siRNA, as indicated by western blot (Figures 4A). As shown in Figures 4A–C, knockdown of ASPP2 significantly increased LC3-II and Beclin1 levels as compared with siRNA control (*p* < 0.05) or untreated control (*p* < 0.01) at low dose gp120. Immunofluorescent results (Figures 4E) showed that the number of autophagy positive cells in ASPP2 siRNA group was significantly increased as compared with that in siRNA control group at 24 h, and the population of cells with autophagy positive characteristics increased significantly in ASPP2 siRNA group than control siRNA group, (35 ± 2.65 and 23.6 ± 2.4%, respectively, *p* < 0.05). In contrast, when SH-SY5Y cells were treated by high dose gp120, both LC3-II and Beclin1 levels (Figures 4F–H) were significantly lower in ASPP2 siRNA than that of siRNA control group (*p* < 0.05). The number of autophagic cells (Figures 4I,J) was significantly lower in ASPP2 siRNA group than that in siRNA control group (22.6 ± 2.6 and 39.7 ± 3.18%, respectively, *p* < 0.05). Taken together, our results indicated that ASPP2 knockdown could promote autophagy at lower gp120 dose but inhibit autophagy at high dose gp120 in SH-SY5Y cells.

In addition, we detected the early apoptotic cells using flow cytometric analysis following staining with AnnexinV/7-aad kit. The results showed that gp120 induced apoptosis (Figure S1) was significantly attenuated by ASPP2 siRNA as compared with siRNA control group (Figures S2A,B, *p* < 0.05).

## Discussion

HIV-1 can inhibit autophagy in cells infected by HIV-1, such as CD4 T cells and macrophages, thereby ensuring viral proliferation (Zhou and Spector, 2008). In contrast, some HIV-1 toxic proteins such as gp120 and Tat can induce autophagy in neurons *in vitro* (Spector and Zhou, 2008). Furthermore, increased markers of autophagy were observed in postmortem brains with HIV-1 encephalitis when compared with that without HIV-1 encephalitis (Zhou et al., 2011), suggesting that autophagy malfunction may be involved in neuroAIDS. Gp120 is a HIV envelope glycoprotein, and it can stimulate pro-apoptotic signal via chemokine receptors (CXCR4 or CCR5) on neurons and microglia in a p53-dependent signaling pathway. Gp120 can increase autophagic proteins and autophagosomes *in vitro* (Hesselgesser et al., 1997; Yeung et al., 1998). P53 is required for the neuronal apoptosis induced by gp120 (Garden et al., 2004).

ASPP2 is a haplo-insufficient tumor suppressor, and can inhibit tumor growth through p53-dependent and -independent pathways in lymphoma, sarcoma, squamous cell carcinoma, and hepatocellular carcinoma (Vives et al., 2006; Kampa et al., 2009; Tordella et al., 2013). ASPP2 also has the function to induce apoptosis by the regulation of autophagy independent of p53 (Wang et al., 2012; Liu et al., 2014; Shi et al., 2015; Xie et al., 2015). In recent years, several studies have shown that ASPP2 has effects on many diseases through regulating autophagy. Xie et al. have found that ASPP2 can attenuate triglyceride to protect against hepatocyte injury by reducing autophagy in cell or mouse model with non-alcoholic fatty liver disease (Xie et al., 2015), and ASPP2 can enhance the apoptosis of oxaliplatin (L-OHP)-induced colorectal cancer cells in a p53-independent manner by inhibiting autophagy (Shi et al., 2015). Liu et al. have also found that ASPP2 can induce autophagic or apoptosis by promoting p53- or p73-independent C/EBP homologous protein (CHOP) expression in hepatoma cells (Liu et al., 2014). In pancreatic cancer, decreased ASPP2 could lead to higher proliferation and autophagic flux of cancer cells, which contributes to the resistance to gemcitabine (Song et al., 2015).

The mechanism that ASPP2 regulates autophagy and apoptosis is still not fully elucidated. The increasing evidence has demonstrated that the molecular structure of ASPP2 may involve in the regulation of autophagy. Based on the molecular structure, the N terminal of ASPP2 is similar to the folded structure of ATG12, one of the key proteins at the initial stage of autophagy. ASPP2 may inhibit autophagy by competing with ATG5 in combination with ATG12 at the initial step of autophagy (Wang et al., 2012). It has been reported that activated RAS can lead to the translocation of ASPP2 from intercellular space to cytoplasm at where ASPP2 combines with ATG5/ATG12 complex, thereby inhibiting autophagy. Hence, ASPP2 can modulate oncogenic RAS-induced autophagic activity to enhance cellular senescence, and inhibit tumor growth (Canning et al., 2012; Wang Z. et al., 2013). Furthermore, ASPP2 can induce the expression of damage-regulated autophagy modulator (DRAM) that cooperates with free Beclin-1 to induce autophagic or apoptosis in hepatoma cells (Liu et al., 2014). Hence, the location of ASPP2 in cells seems to have relationship with its regulation of autophagy and apoptosis. ASPP2 can also be transferred to the nucleus and combined with p53, to play its role in stimulating p53 and promoting apoptosis under stress condition (Liu et al., 2016).

In this study, we found that gp120 increased autophagic proteins Beclin1 and LC3-II level at a dose-dependent manner in neuroblastoma cell line SH-SY5Y. ASPP2 can significantly inhibit autophagy and apoptosis in the presence of low dose of gp120 and promote autophagy and apoptosis at high dose of gp120. These results suggested that ASPP2 has a dual regulatory role in autophagy and apoptosis in the presence of gp120 with different concentrations. In our previous study, we had found the interaction of ASPP2 with p53 induced by gp120 in the primary cerebrocortical neuron. In that study, we found that gp120 protein (30 ng/mL) could stimulate translocation of both p53 and ASPP2 to the nucleus, leading to Bax transcription and caspase-3 cleavage (Liu et al., 2016). When treated in low dose of gp120, ASPP2 located at cytoplasm and inhibited autophagy via competing with ATG5 in combination with ATG12 at the initial step of autophagy. Then when treated by high dose of gp120, ASPP2 was translocated to the nucleus and induced autophagy and apoptosis through p53 pathway. Combined with above results, we can conclude that the concentration of gp120 affects the location of ASPP2 which determines the effect of ASPP2 on autophagy and apoptosis.

In summary, for the first time the effect of ASPP2 on autophagy and apoptosis induced by gp120 were investigated *in vitro*. We found that ASPP2 had different effects on the autophagy and apoptosis of neurons induced by different concentration of gp120 protein. Thus, ASPP2 probably play an important role in HAND or HAD, It may be a potential therapeutic agent for HAND through modulating autophagy and apoptosis in CNS, but the actual effects and its underlying mechanisms need to be further explored.

## Author contributions

ZL, LQ, HW, and DC conceived the study, designed the experiments, and analyzed the data. ZL, LQ, YJZ, XL,YLZ, XZ, and YS performed the experiments, YS, XZ, KL, YLZ, BS, LY, and TZ contributed to reagents and materials; ZL, QL, XL, KL, BS, YJZ, HW, and DC wrote the article. All authors read and approved the final manuscript.

### Conflict of interest statement

The authors declare that the research was conducted in the absence of any commercial or financial relationships that could be construed as a potential conflict of interest.

## Supplementary material

Figure S1 **Detection of early apoptosis using the flow cytometric analysis following staining with AnnexinV/7-aad kit**. Early apoptotic cells that stain with Annexin V-PE only are located in the lower right quadrant of three displays.

Figure S2 **(A)** Detection of apoptotic cells occurred by flow cytometric analysis following staining with AnnexinV/7-aad kit. **(B)** The mean levels of early apoptosis cells in different groups. ^*^P < 0.05; ^**^P < 0.01.
