# Supplementary figures and images for: ASPP2 Plays a Dual Role in gp120-Induced Autophagy and Apoptosis of Neuroblastoma Cells
Source: Front Neurosci. 2017 Mar 24;11:150. doi: 10.3389/fnins.2017.00150 (PMC5364170; doi:10.3389/fnins.2017.00150)

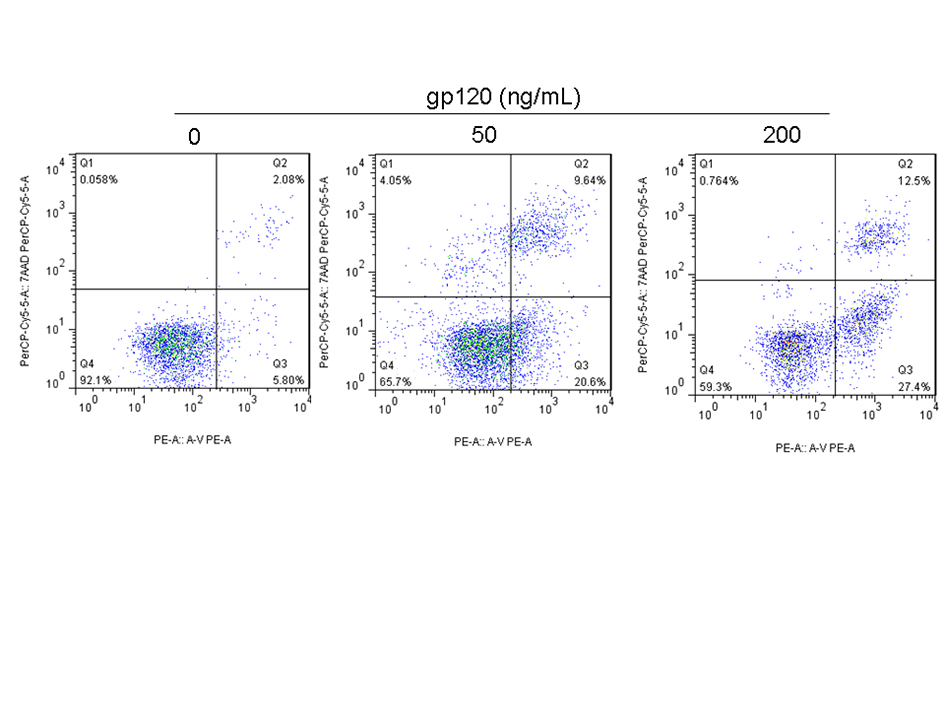

Supplement: Figure S1 — Detection of early apoptosis using the flow cytometric analysis following staining with AnnexinV/7-aad kit. Early apoptotic cells that stain with Annexin V-PE only are located in the lower right quadrant of three displays. [file Image1.TIF]

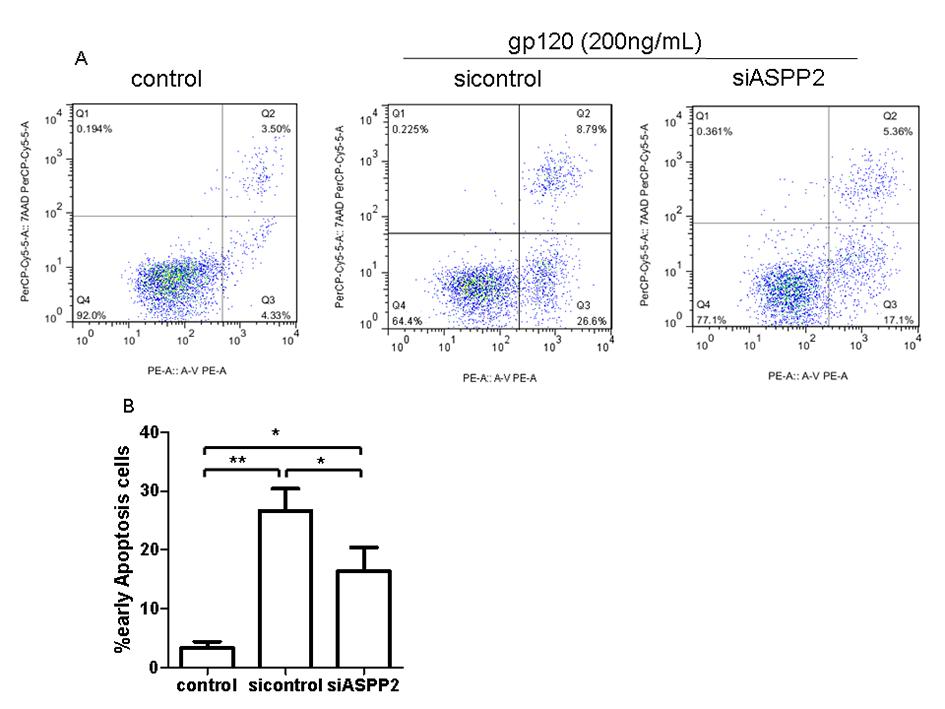

Supplement: Figure S2 — (A) Detection of apoptotic cells occurred by flow cytometric analysis following staining with AnnexinV/7-aad kit. (B) The mean levels of early apoptosis cells in different groups. *P < 0.05; **P < 0.01. [file Image2.TIF]
